# Knowledge and Practice of Kerman Dentists about Infection Control in Digital Radiography

**DOI:** 10.30476/DENTJODS.2021.91381.1578

**Published:** 2022-12

**Authors:** Jahangir Haghani, Hamed Ebrahimnejad, Molook Torabi-Parizi, Marzieh Karimi-Afshar, Roya Amiri

**Affiliations:** 1 Dept. of Oral and Maxillofacial Radiology, Oral and Dental Diseases Research Center, Kerman University of Medical Sciences, Kerman, Iran; 2 Social Determinants on Oral Health Research Center, Kerman University of Medical Sciences, Kerman, Iran; 3 Dept. of Orthodontics, School of Dentistry, Kerman University of Medical Sciences, Kerman, Iran; 4 General Dentist, Kerman, Iran

**Keywords:** Awareness, Dental radiology, Infection control

## Abstract

**Statement of the Problem:**

Infection control is essential for a safe clinical environment during patients’ treatment in dentistry. Transmission of the infection can occur due to contact with patients’ saliva and blood in radiology clinics.

**Purpose:**

The purpose of this study was to investigate the knowledge and practice of dentists in Kerman about infection control in digital radiology.

**Materials and Method:**

This cross-sectional study was conducted on 162 dentists who worked either in private office or clinic or both. The samples were selected through simple sampling method. Data were collected through demographic data, and valid and reliable questionnaire consisted of 7 questions about knowledge and 12 questions about practice on infection control in digital radiology. Data were analyzed in SPSS 26 software using T, ANOVA, and linear regression tests. The *p* value was considered at 0.05% significant level.

**Results:**

86(53.1%) of participants were men and 76 (46.9%) were female. The mean age of participants and work experience were 36.32±8.88 and 11.03±8.53 years, respectively. The mean and standard deviation of knowledge, practice, and total were 5.20±1.26, 7.98±2.00, and 13.22±2.72, respectively. There was a positive significant correlation between knowledge with age of participants (*p*= 0.009). There was also a direct significant correlation between knowledge and practice with work experience (*p*= 0.045 and *p*= 0.01 respectively).

**Conclusion:**

Knowledge and practice of dentists in Kerman about infection control in digital radiology were good and medium respectively. However, there was a direct significant correlation between knowledge and practice of dentists. Knowledge and practice scores in dentists who worked in private office were significantly better than those who worked only in clinics.

## Introduction

Dental radiographic appliances are a potential infection source because of contamination with saliva and blood; therefore, the risk of cross-contamination in dental radiology is high [ 1 - 2 ]. Today, digital radiographs are widely used due to their many advantages [ 3 ]. Therefore, due to the increasing use of digital radiography and lack of study about infection control in digital radiography in Iran, we designed a study about knowledge and practice of dentists in Kerman on the principles of infection control in digital radiology. 

## Materials and Method

This cross-sectional study (IR code. KMU. REC. 1396. 1496), was approved by the ethics committee of Kerman University of Medical Sciences. In this study, 162 randomly selected dentists were participated in 2019. The sample size was determined according to the population of employed dentists and similar studies. Data were collected by a employing a questionnaire ,which was prepared based on Centers for Disease Control and Prevention (CDC) and articles in this field [ 1 , 4 - 6 ] and its validity was confirmed by 5 faculty members (Content validity index= 0.78). Its reliability was also accepted through test-re-test (20 dentists were completed the questionnaire twice in 3 weeks interval).The intraclass correlation coefficient was set at 0.85.

The questions consisted of three parts including (1) demographic information, (2) knowledge (7 questions), and (3) practice (12 questions). The range score knowledge and practice was 0-7 and 0-12 respectively. Each correct response was given score ‘one’ and wrong answers were given score ‘zero’. The scores of knowledge were classified between 0 and 2.3 as poor, 2.4to 4.7 as medium, and 4.8to 7 as good. The scores of practice were classified between 0 and 4 as poor, 4.1 to 8 as medium, and 8.1to 12 as good. In order to complete the questionnaires, a trained student referred to dental clinics and offices and after obtaining the consent, questionnaires were completed by dentists in the same session.

SPSS 26 program was used for statistical analysis. T-test, ANOVA, and linear regression were used to analyze the data at a significance level of 0.05%.

## Results

Of all participants, 86 (53.1%) were male. The mean age and work experience was 36.32±8.88, and 11.03 ±8.53 respectively. Of the total, 24.1% were employed in the office, 41.4% in the clinic, and 34.5% in both places. Of 162, 71.6% had a digital radiography device at their workplace, 84.0% had trained their staff, and 25.3% were participated in infection control courses. 

**Figure 1:**
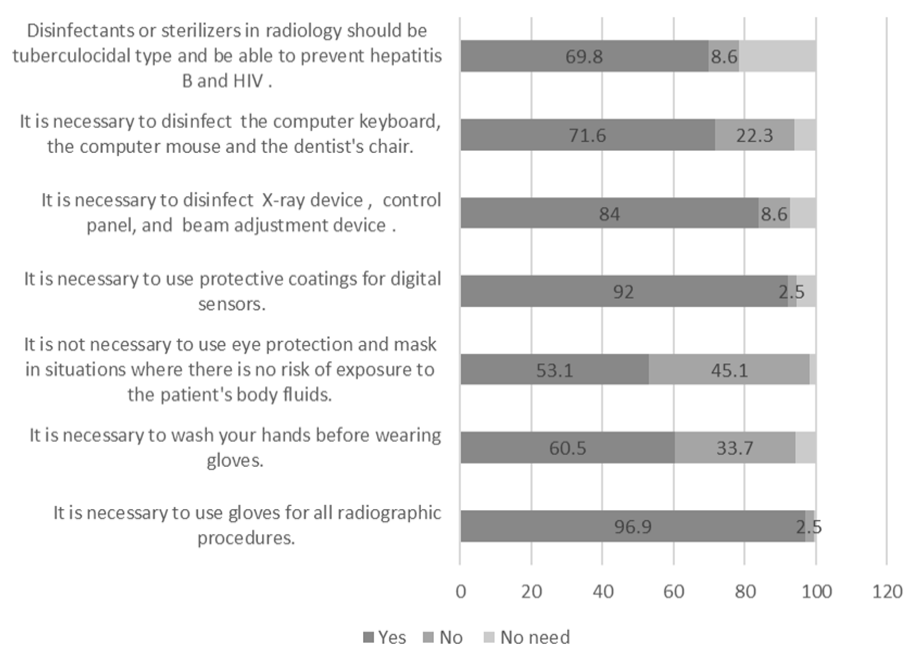
Items of knowledge and percentage of answers to these questions

In knowledge aspect, the highest percentage of correct answers was related to item " It is necessary to use gloves for all radiographic procedures” and the lowest percentage of correct answers was related to item " It is not necessary to use eye protection and mask in situations where there is no risk of exposure to the patient's body fluids” (Figure 1).

**Figure 2:**
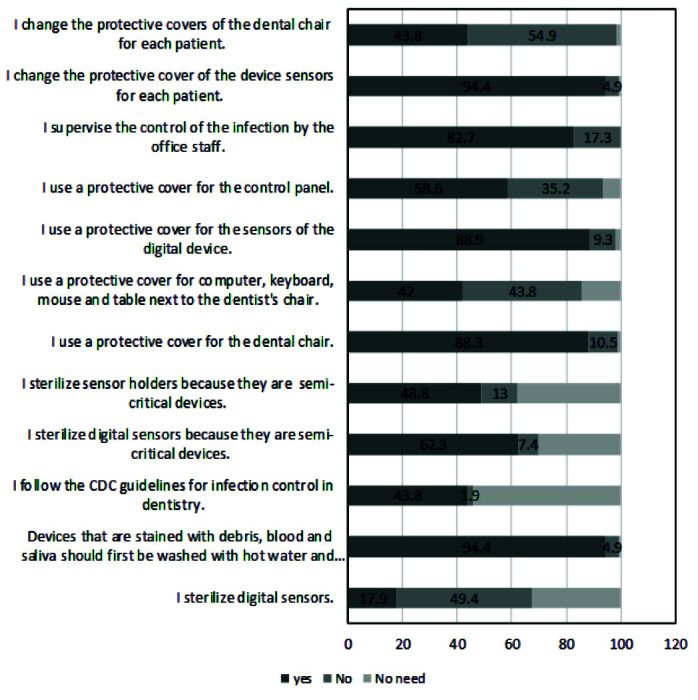
Items of practice and percentage of answers to these questions

In practice area, the highest percentage of correct answers (94.4%) was related to two items including 1: " Devices that are stained with debris, blood, and saliva should first be washed with hot water and soap and then sterilized” and 2: “I change the protective cover of the device sensors for each patient". The lowest percentage of correct answers (42%) was related to item “I use a protective cover for computer, keyboard, mouse and table next to the dentist's chair” (Figure 2).

The mean of score knowledge and practice was found to be 5.20±1.26 (76.5=good, 19.1%=medium, 4.3%= poor) and 7.98±2.0 (43.2%=good, 53.7%=medium, 3.1 %= poor) respectively. The mean total score was 13.22± 2.72 (35.9%=good, 63.0%=medium, 1.2%= poor). 

The knowledge and practice of people who worked only in the office were significantly higher than dentists who worked only in clinic (*p*= 0.010, *p*= 0.013 and *p*= 0.002), respectively. There was not a significant relationship between knowledge and practice and total scores with sex, having a digital radiography, infection control education and participating in infection control courses (*p*> 0.05). 

The multivariate analysis showed that there was a statistically significant relationship between age and knowledge (B= 0.26, *p*= 0.009) and total score (B=0.2, *p*= 0.02), but there was no statistically significant relationship between age and practice (*p*= 0.09). In addition, there was a significant positive relationship between knowledge with practice (B= 0.34, *p*= 0.0001) and work experience (B= 0.16, *p*= 0.045).

## Discussion

This study showed that knowledge and practice of dentists were good and medium respectively. However, there was a positive significant correlation between dentists’ knowledge and practice.

Many previous studies [ 1 - 5 ] focused on the importance of infection control in dental radiography. In some studies, the knowledge and practice of dentists and dental students about infection control in conventional radiographs has been evaluated. For example, in a study conducted by Haghnegahdar *et al.* [ 1 ] in Shiraz, the knowledge of general dentists about infection control was poor during the preparation of intraoral radiography, which was lower than the findings of the present study [ 1 ]. However, we evaluated knowledge and practice of dentists about infection control in digital radiography. 

Digital intraoral radiographs, unlike X-ray films, use digital sensors, which are sensitive to disinfectants and cannot be sterilized by autoclaves. Therefore, the use of surface barriers is essential to prevent contamination [ 4 ]. In our study, 62.3% of dentists classified digital sensors as semi-critical instruments and covered by protective barriers. This percentage was higher than the results (51.45%) of Alakhras *et al.* [ 5 ]. In addition, in present study, 9.3% of dentists did not use a protective cover for the sensors and 35.2% for control panel. It has been shown that there is a possibility of transmitting the infection during use and processing and disinfection of phosphor plates (PSP) [ 6 ]. PSP plates should be disinfected after removing a contaminated surface barrier and the barriers should be kept in an aseptic environment. In addition, these coatings should be carefully evaluated before use [ 6 ] and using two barriers can be helpful in preventing rupture of intraoral barriers [ 3 ].

Concerning personal protective equipment (PPE), in this study, 96.4% of dentists knew that gloves should be used in all stages and 60.5% knew that it is necessary to wash hands before wearing gloves. In addition, 53.1% knew that eye protection was necessary in all stages of radiography. In Anders *et al.*'s study [ 5 ], 82% of dental students used eye protection at all stages. Improper use of the mask was seen in 24% of people, and improper use of gloves was observed in 35% of cases. Moreover, 83% of subjects washed their hands before using gloves. In a study by Friere *et al.* [ 7 ], improper use of gloves and mask was observed in 8% and 38% of dental students respectively. The differences observed in these studies with our study may be due to the fact that the current study was conducted by a questionnaire, which completed by the participants, while in the study by Anders *et al.* [ 5 ] and Freire *et al.* [ 7 ], practice of dental student was observed. 

In this study, dentists who worked in the office had significantly better knowledge and practice. This can be explained as fewer and patients who are more sensitive refer to the office, which may subsequently prompt dentists to control the infection more accurately. In addition, 84% of the dentists had trained their staff on infection control. In the study by Lima *et al.* in Brazil, the assistants had not received any training [ 8 ]. 

In our study, a positive and significant correlation was observed between knowledge and practice in the field of infection control in digital radiography. The studies show that practice may be affected by many factors such as knowledge and educational background, sociodemographic and professional variables, and access to required materials and equipment [ 9 - 10 ]. However, although the awareness in our study results was good, the practice was scored medium. About 43.8% of the dentists did not control the infection according to the CDC guidelines; this finding was similar with the results of a study by Dagher *et al.* [ 4 ] in Lebanon. 

We found there was a marginal significant relationship between participation in infection control courses and practice. It seems that participation in continuing education courses is an important factor for improving of practice in infection control. 

Our results showed that increasing age and work experience had a positive significantly relationship with knowledge and practice. This result is consistent with a study by Dagher *et al.* in Lebanon [ 4 ], which presented that dentists in practice for more than 20 years showed more compliance with hands hygiene and use of PPE. However, it is inconsistent with the studies of Ghasemi *et al.* [ 11 ] In Iran and Al-Rabeah and Mohamed [ 12 ] in Saudi Arabia, which showed young dentists, had better practice. It seems that although more experienced older dentists may have little knowledge about infection control during their graduate years, in some cases, they can gain considerable knowledge during their professional experience. 

As the limitation of this study, the information about knowledge and practice were collected by questionnaire, which may consequently have been over reported.

## Conclusion

This study showed that knowledge and practice of dentists in Kerman about infection control in digital radiology were good and medium, respectively. Further studies should be designed to evaluate knowledge and practice of dentists and dental students about control infection in radiology. 

## Acknowledgement

This work was supported by Oral and Dental Diseases Research Center, Kerman University of Medical Sciences, Kerman, Iran.

## Conflict of Interest

 The authors declare that they have no conflict of interest.
